# Vibrational Spectroscopy Through Time Averaged Fourier Transform of Autocorrelated Molecular Dynamics Data: Introducing the Free SEMISOFT Web‐Platform

**DOI:** 10.1002/jcc.70118

**Published:** 2025-05-03

**Authors:** Riccardo Conte, Michele Gandolfi, Davide Moscato, Chiara Aieta, Stefano Valtolina, Michele Ceotto

**Affiliations:** ^1^ Dipartimento di Chimica Università degli Studi di Milano Milano Italy; ^2^ Dipartimento di Informatica “Giovanni Degli Antoni” Università degli Studi di Milano Milano Italy

**Keywords:** free web‐platform, power spectra, theoretical spectroscopy, theoretical vibrational dynamics, time averaged Fourier transform

## Abstract

Vibrational spectroscopy calculations based on classical molecular dynamics simulations are widely employed in a variety of popular fields, for instance, computational biochemistry and materials science. These calculations commonly rely on the Fourier transform of the velocity autocorrelation function. One major drawback of the method is that calculated spectra are difficult to interpret due to the large number of closely spaced signals. In this paper, we show how theory can help to overcome this issue by means of a time‐average technique, and we introduce free software to perform such calculations for anyone who may take advantage of it. The studies presented here involve the classical vibrational spectra of aniline microsolvated by a water molecule and gas‐phase deoxyguanosine. The software is made available in the form of a free web‐platform, named SEMISOFT (http://semisoft.unimi.it/), whereby, upon upload of the classical trajectory, the user gets the corresponding time‐averaged spectrum. Furthermore, since the evaluation of response functions through autocorrelated data is quite a general approach, the web‐platform can be directly employed in many other research fields besides chemistry.

## Graphical abstract

A free web‐platform for the production of spectra based on data collected from classical molecular dynamics runs is introduced. Its effectiveness is demonstrated through the calculation of the vibrational spectra of monohydrated aniline and deoxyguanosine. The platform is easy to use and based on the time‐averaged Fourier transform of autocorrelated data, a general approach that may be adopted in several fields.

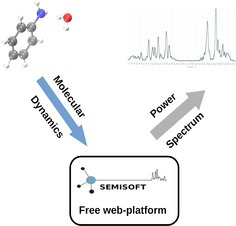

## 1 Introduction

Molecular dynamics (MD) is a powerful computational technique that can be employed in a variety of applications. For instance, thermodynamical functions can be determined by means of a molecular dynamics run. Likewise, structural properties are obtained from molecular dynamics simulations: The radial distribution function, commonly used to get a measure of the local structure of a solvated system, is one of them. In general, from MD simulations, one can calculate correlation functions, and from there get an estimate of transport properties and a detailed description of spectroscopic features [1, 2].

Molecular dynamics and correlation functions are amenable to both classical and quantum mechanical investigations. The latter provides the correct chemical description of the system, while the former are much computationally cheaper and often captures a good amount of physical insight. As a consequence, in the field of theoretical chemistry, research is still aiming at improving high‐level but computationally affordable theories able to accurately approximate quantum mechanics for application to large dimensional or complex molecular systems [3, 4, 5, 6, 7, 8, 9, 10, 11, 12, 13, 14, 15, 16]. Trying to tackle the issue with a completely different approach, many efforts are also being made in the field of quantum computing, with the aim of directly performing quantum calculations on a tailored quantum infrastructure [17, 18, 19]. In this latter case, applications have so far been limited to low‐dimensional systems due to technical difficulties in overcoming decoherence phenomena. Conversely, classical calculations, including classical MD, can be straightforwardly implemented into traditional computers and applied to large systems [20].

Among the many different applications, it is particularly interesting that classical MD is employed for spectroscopic calculations. The theory of spectroscopy is based on quantum mechanics, and under the collective “Spectroscopy” name, one can find several techniques able to detect transitions between quantum energy levels. However, if we focus, for instance, on vibrational spectroscopy, the estimates of the classical frequencies of vibration are directly comparable with the fundamental frequencies anticipated by quantum mechanics. To some degree of approximation, the adoption of classical calculations is therefore possible also in the field of vibrational spectroscopy. A more accurate approach, based on classical trajectories, is the quasi‐classical trajectory (QCT) one [21, 22]: In QCT, the initial conditions of the trajectory are harmonically quantized (zero‐point energy is included) but the evolved trajectory is fully classical, so QCT neglects quantum interference and other possible nuclear quantum effects. Therefore, QCT is able to account for spectral anharmonicity only to a certain degree [20, 23, 24]. To close even more the gap between classical and quantum spectroscopy simulations, several techniques based on classical trajectories and able to reproduce at least approximately nuclear quantum effects are being developed. Popular examples of this are semiclassical dynamics [25, 26, 27, 28, 29, 30, 31, 32, 33, 34, 35, 36, 37], or path‐integral molecular dynamics (and methods derived from it) [13, 38, 39].

In this manuscript, we focus on linear spectroscopy, specifically on the calculation of spectra based on Fourier transforming autocorrelated data. The theory of linear response applies to both classical and quantum physics in the presence of a weak perturbation [40]. It represents a way to evaluate how an equilibrium system changes in response to an external perturbation. The quantity associated with this phenomenon is called a response function, and it is a real observable quantity. It turns out that response functions can be expressed in terms of time correlation functions, which can be computed starting from molecular dynamics runs. Furthermore, the perturbed system is in nonequilibrium, but it is the equilibrium fluctuations that determine the nonequilibrium response, so MD is conveniently run for the unperturbed system.

The main issue of a method based on the Fourier transforming of autocorrelated data is that when the method is applied to large‐dimensional systems, the spectrum is crowded with signals. Of course, this is true also for the corresponding experimental spectra, so there is a clear need for a computational approach characterized by the following three features: (i) being based on classical MD for application to large systems granting atomistic detail and at least partial description of the anharmonicity in the vibrational spectrum; (ii) allowing one to selectively assign spectroscopic features; (iii) being general and not dependent on the type of system or on the chosen level of electronic theory. Even if these three requirements are apparently easy to be meet, this is often not the case. For instance, the first and, most of all, the third point do not apply to scaled harmonic methods [41]: A different scaling coefficient applies to different levels of electronic structure theory. Such an *ad hoc* approach, even if very practical, has to be avoided to come up with a correct assignment and interpretation of the spectrum.

In this paper, we start by showing that classical frequencies of vibration can be obtained from the Fourier transform of the velocity autocorrelation function. Then, we move to normal mode coordinates and apply a time averaging technique. The final formula has the advantage of separating the several vibrational modes, making the spectrum more readable and the signals assignable. The calculations are performed on a free web‐platform, whose main features are described in the dedicated Subsection of the Methodology Section. The Results Section will show three different simulations performed on two chemical systems. They are aniline microsolvated by a single water molecule (calculations involve both Cartesian and normal‐mode dynamics) and deoxyguanosine (calculation only in Cartesian coordinates). Some conclusions end the paper.

## 2 Methodology

### 2.1 The Fourier Transform of the Velocity Autocorrelation Function: A Way to Calculate Classical Vibrational Frequencies

IR spectroscopy is based on the interaction between the electromagnetic radiation (in the IR frequency domain) and matter. In absorption spectroscopy, when the energy of the radiation matches the energy gap between two differently populated vibrational states of a molecule, a photon is absorbed, and the corresponding vibration gets excited. This quantum picture of the phenomenon can find, at least when the focus is on fundamental frequencies, an analogous classical picture. If $H_{0}$ indicates the classical Hamiltonian of the isolated molecule, the external electric field E (generated by the IR experimental spectrometer) is a perturbation of the isolated system. If the field resonates with the molecular dipole, then the molecule can absorb energy and vibrate more energetically. When the perturbation is weak, the response of the molecule depends linearly on it, and the Hamiltonian is

(1) $$H^{\prime }=H_{0}+\lambda H_{1}$$

where $H_{1}$ is the perturbation induced by the external field, and λ is a dimensionless scalar parameter. For weak perturbations, Equation (1) is taken in the λ→0 limit.

To come up with a working formula, we start from the classical definition of the partition function in a canonical ensemble at temperature T

(2) $$Q=\frac{1}{h^{3N}}\int e^{-\beta H_{0}}d\Gamma$$

where $d\Gamma =d{\text{p}}_{0}d{\text{q}}_{0}$ is the volume element in phase space, and $\beta =1/k_{B}T$, $k_{B}$ being the Boltzmann constant. h, usually chosen to be Planck's constant, is an action term which makes the partition function of the energetically continuous classical system adimensional, and N is the number of atoms in the molecule. Given the partition function Q, it is possible to calculate the Boltzmann average

(3) $$\langle O\rangle =\frac{1}{Q}\int \;e^{-\beta H_{0}}\;O\;d\Gamma$$

of any observable O. Analogously, if we move to the partition function for the perturbed system, we get

(4) $$Q^{\prime }=\frac{1}{h^{3N}}\int e^{-\beta (H^{\prime })}d\Gamma =\frac{1}{h^{3N}}\int e^{-\beta (H_{0}+\lambda H_{1})}d\Gamma$$

and the observable average for the perturbed system (${\left\langle O\right\rangle }^{\prime }$) is

(5) $${\left\langle O\right\rangle }^{\prime }=\frac{1}{Q^{\prime }}\int \;e^{-\beta (H_{0}+\lambda H_{1})}\;O\;d\Gamma$$

Now let's consider the case, fitting our final goal, in which the perturbation is small enough that it can be considered negligible in the expression of the partition function $Q^{\prime }.$ This means that we approximate $Q^{\prime }\approx Q$. Conversely, we keep the perturbation in the integrand of ${\left\langle O\right\rangle }^{\prime }$ and rewrite it according to

(6) $$\begin{array}{ll} {\left\langle O\right\rangle }^{\prime } & \approx \frac{1}{Q}\int \;e^{-\beta H_{0}}e^{-\beta \lambda H_{1}}\;O\;d\Gamma \\ & \approx \frac{1}{Q}\int \;e^{-\beta H_{0}}(1-\beta \lambda H_{1})\;O\;d\Gamma \\ & =\langle O\rangle -\frac{\beta \lambda }{Q}\int \;e^{-\beta H_{0}}\;H_{1}\;O\;d\Gamma \\ & =\langle O\rangle +\lambda \langle \Delta O\rangle \; \end{array}$$

In Equation (6), based on the fact that λ→0, the following (approximate) linearization has been adopted

(7) $$e^{-\beta \lambda H_{1}}\approx 1-\beta \lambda H_{1}$$

$\left\langle \Delta O\rangle =-\beta \langle H_{1}O\right\rangle$. In case $H_{1}$ is the interaction between the molecular charge distribution and an electric field in the IR domain, calculation of ⟨ΔO⟩ leads to the IR spectrum of the molecule.

We now proceed to rearrange ⟨ΔO⟩ in a more manageable form by focusing on the case of IR spectroscopy. One way to look at the effect of the perturbation is to follow the system during its relaxation after the perturbation is terminated. We exploit the system's ergodicity to substitute the equilibrium ensemble average (i.e., based only on the $H_{0}$ Hamiltonian) with a time average, in which the perturbation is taken at t=0 and the observable O is calculated at a time t after the perturbation has been switched off. Such calculations can be performed by introducing the following general correlation function

(8) $$C(t)=\frac{1}{T}\int_{0}^{T}\;\langle H_{1}(t_{0})\;O(t_{0}+t)\rangle \;dt_{0}$$

The molecular dynamics must be sufficiently long and performed on the unperturbed system since the original ensemble average in Equation (6) is based on an equilibrium distribution. The ⟨⟩ symbol now indicates that the average over the three Cartesian coordinates (and, when required by the observable, over all atoms) must be taken.

In IR spectroscopy, the observable one focuses on is O=E(0)·μ(t). It is the term $H_{1}$ which takes into account how the external field perturbs the molecule, that is, $H_{1}=-\mu (0)\cdot \text{E}(0)$. Upon averaging over the polarization directions of the radiation, $\left\langle H_{1}O\right\rangle$ reduces to ⟨−μ(0)·μ(t)⟩ [42]. Even if the absolute absorption intensity of the IR spectrum further depends on variables like the temperature and intensity, and frequency of the external field, just by calculating the Fourier transform of the oscillating $C_{\mu \mu }$ function, one gets the absorption lineshape from which the relative absorption intensities can be obtained. Furthermore, this provides an accurate classical estimate of the frequencies of vibration, which contain most of the information one wants to collect from a calculated spectrum [43]. So, for $C_{\mu \mu }$ it is possible to write

(9) $$C_{\mu \mu }(t)=\frac{1}{T}\int_{0}^{T}\;\langle \mu (t_{0})\mu (t_{0}+t)\rangle \;dt_{0}$$

From the relation $C_{\mu \mu }(t)\propto \langle \Delta \mu (t)\rangle$ it descends the well‐known fact that the IR‐active vibrations are those for which the absorption of the photon is associated to a molecular motion that produces a variation of the molecular dipole. As anticipated, the time T, that is, the total evolution time of the dynamics, is chosen sufficiently large. In the case of vibrational spectroscopy, the dynamics of the dipole must be calculated for a time long enough to allow the molecule to perform a large number of vibrations. Furthermore, MD simulations provide atomic positions and velocities, so by noticing that the dipole can be linearized as a linear function of the spatial coordinates q, it is possible to move from the $C_{\mu \mu }$ to the $C_{qq}$ autocorrelation function without loss of information about the frequencies. Historically, the basically equivalent $C_{vv}$ ($\text{v}=\dot{\text{q}})$ autocorrelation function has been more commonly employed.

So far, we have defined autocorrelation functions in the Cartesian coordinates. They certainly provide features i) and iii) required in the Introduction for a general computational spectroscopy approach. However, the Cartesian autocorrelation functions account for all frequencies of vibrations, and the resulting Fourier transform yields a spectrum as crowded as the experimental one. Requirement ii) can be met if we move from the Cartesian to the normal mode coordinates, and this is doable by means of a linear transformation. In the case of velocities, the transformation leads to normal‐mode momenta, and the autocorrelation is named $C_{pp}$. In a non‐linear molecule, there are 3N−6 normal modes, so one may compute 3N−6 autocorrelations, one per each mode. By Fourier transforming the j‐th of these $C_{pp}$ autocorrelation functions, only signals relative to the j‐th mode or modes strongly coupled to it are obtained. In this way, it is possible to abide by the three criteria illustrated in the Introduction.

### 2.2 Exploiting the Time Average: An Approach to Get Positive‐Definite Spectra

It is possible to exploit the time average in Equation (8) to come up with a more convenient way to calculate classical vibrational frequencies. We start from the expression of the Fourier transform of the $C_{pp}$ autocorrelation function for the j‐th normal mode

(10) $$I_{j}(\omega )=\int_{-\infty }^{+\infty }e^{i\omega t}\frac{1}{T}\int_{0}^{T}p_{j}(t_{0})p_{j}(t_{0}+t)dt_{0}dt$$

where $I_{j}(\omega )$ is known as the power spectrum relative to mode j, and we exploit the Fourier anti‐transforms of $p_{j}(t_{0})$ and $p_{j}(t_{0}+t)$

(11) $$p_{j}(t_{0})=\frac{1}{2\pi }\int_{-\infty }^{+\infty }\;e^{-i\omega^{\prime }t_{0}}{\tilde{p}}_{j}(\omega^{\prime })d\omega^{\prime }$$

(12) $$p_{j}(t_{0}+t)=\frac{1}{2\pi }\int_{-\infty }^{+\infty }\;e^{-i\omega^{\prime \prime }(t_{0}+t)}\tilde{p_{j}}(\omega^{\prime \prime })d\omega^{\prime \prime }$$

The simulation time T is chosen sufficiently long to sample the vibrational motions, so with negligible approximation, we can set the upper limit of the $t_{0}$‐integration to +∞. Equation (10) can be recast as

(13) $$\begin{array}{c} \begin{array}{l} I_{j}(\omega )=\frac{1}{{\left(2\pi \right)}^{2}T}\int_{-\infty }^{+\infty }\int_{-\infty }^{+\infty }\int_{-\infty }^{+\infty }\;e^{i(\omega -\omega^{\prime \prime })t}\\ \;\times \int_{0}^{+\infty }\;e^{-i(\omega^{\prime }+\omega^{\prime \prime })t_{0}}\;dt_{0}\;dt\;{\tilde{p}}_{j}(\omega^{\prime })\;{\tilde{p}}_{j}(\omega^{\prime \prime })\;d\omega^{\prime }\;d\omega^{\prime \prime }\\ \;=\frac{1}{{\left(2\pi \right)}^{2}T}\int_{-\infty }^{+\infty }\int_{-\infty }^{+\infty }\int_{-\infty }^{+\infty }\;e^{i(\omega -\omega^{\prime \prime })t}(\pi )\\ \;\times \delta (-(\omega^{\prime }+\omega^{\prime \prime }))\;dt\;{\tilde{p}}_{j}(\omega^{\prime })\;{\tilde{p}}_{j}(\omega^{\prime \prime })\;d\omega^{\prime }\;d\omega^{\prime \prime }\\ \;=\frac{1}{(2\pi )2T}\int_{-\infty }^{+\infty }\int_{-\infty }^{+\infty }e^{i(\omega -\omega^{\prime })t}\;dt\;{\tilde{p}}_{j}(\omega^{\prime })\;{\tilde{p}}_{j}(-\omega^{\prime })\;d\omega^{\prime }\; \end{array} \end{array}$$

In Equation (13), we take advantage of the parity property of the Dirac delta and perform the integration over $\omega^{\prime \prime }$. Similarly, the integration over t will provide a Dirac delta that simplifies the integration over $\omega^{\prime }$

(14) $$\begin{array}{c} \begin{array}{l} I_{j}(\omega )=\frac{1}{(2\pi )2T}\int_{-\infty }^{+\infty }(2\pi )\delta (\omega -\omega^{\prime })\;{\tilde{p}}_{j}(\omega^{\prime })\;{\tilde{p}}_{j}(-\omega^{\prime })\;d\omega^{\prime }\\ \;=\frac{1}{2T}{\tilde{p}}_{j}(\omega ){\tilde{p}}_{j}(-\omega )\\ \;=\frac{1}{2T}{\tilde{p}}_{j}(\omega ){\tilde{p}}_{j}^{*}(\omega )\\ \;=\frac{1}{2T}|{\tilde{p}}_{j}(\omega ){|}^{2}\; \end{array} \end{array}$$

In Equation (14) the equality ${\tilde{p}}_{j}(-\omega )={\tilde{p}}_{j}^{*}(\omega )$ holds because the momentum is real‐valued. The final result is a power spectrum defined by means of a positive‐definite quantity [44]. The frequencies of vibration are located at the frequencies corresponding to peak maxima. If the j‐th mode is not strongly coupled to any other mode, then a single dominant peak will appear in the power spectrum; otherwise, a set of peaks will show up. The peaks due to coupling are typically slightly shifted from the frequency one finds when estimating the corresponding modes. The reason for this is that if mode i is coupled to mode j, then $I_{j}(\omega )$ will present the peak relative to mode j but also a peak relative to mode i, which is estimated slightly differently from what is obtained in the calculation of $I_{i}(\omega )$. However, Equation (14) provides a way to get vibrational frequencies starting from a molecular dynamics run, it works in a general and non‐ad‐hoc fashion, and it largely overcomes the experimental issue of overcrowded spectra.

### 2.3 The Free SEMISOFT Web‐Platform to Calculate Time‐Averaged Fourier Transforms of Autocorrelated Data

Equation (14) and its Cartesian analog involving Cartesian velocities are at the heart of the free SEMISOFT web‐platform that we developed and recently released to provide the scientific community with an easy‐to‐use tool for calculating time‐averaged power spectra starting from the output of a molecular dynamics run [40]. We notice that the formalism of autocorrelation functions as a means to evaluate linear responses to perturbations is quite general and, therefore, the web‐platform can be of general utility. We'll discuss more on this in the Conclusions Section.

Relatively to the vibrational spectroscopy field, the only input the user must provide is an MD simulation. This commonly consists of the set of atomic positions and velocities evolved in time along the trajectory. Initial conditions, from which the MD dynamics processed by SEMISOFT starts, are usually generated according to a quasi‐classical quantization. According to the user's simulation goals, they can also be obtained after an equilibration run in the presence of a thermostat. MD simulations rely on the Born‐Oppenheimer approximation and can be performed by employing dedicated software, which is characterized by the way the electronic problem is tackled. There are basically 3 main ways to generate an MD run.

The first one is through ab initio “on‐the‐fly” molecular dynamics: This technique consists in solving the quantum electronic problem (this is the ab initio part) at each step along the classical dynamics (this is the meaning of “on‐the‐fly”). The electronic structure calculations are performed at a chosen level of theory (DFT and affordable post‐Hartree Fock methods are the most common selections). Ab initio “on‐the‐fly” MD is the most accurate of the three approaches, but it has the drawback of being the slowest when it comes to generate the dynamics. NWChem [45], Orca [46], Quantum Espresso [47], COBRAMM [48], and QCHEM [49] are just some examples (among many others) of software that allow the user to perform ab initio “on‐the‐fly” MD.

The second approach adopts an ab initio analytical potential energy surface (PES) for the system of interest. This is a still‐growing field which has attracted a lot of attention in recent years from the machine learning community [50, 51, 52, 53, 54, 55, 56, 57]. The advantages of this approach are that the level of accuracy is close to that of an ab initio “on‐the‐fly” dynamics and the dynamics is much faster to calculate. However, each different system needs its own PES and the fitting procedure to obtain a high‐level analytical PES can be quite elaborate, and it is far from being a black‐box protocol.

Finally, the third possible kind of approach consists of running the molecular dynamics using a classical approximation to the electronic problem. This is done by means of the so‐called force fields, which are widely employed to study biochemical systems. Here, the advantage is that the dynamics is extremely fast to compute and, therefore, even very large systems can be studied. Furthermore, force fields are in part portable, and the same force field can be used for several molecules of similar chemical composition. The main disadvantage consists in the much lower accuracy compared to the previous two approaches, which, especially in the field of spectroscopy, can be dramatic. Many force fields have been created over the years. For the non‐expert user we cite a few of the perhaps most popular ones without claim of being exhaustive: Allinger MM [58], Amber [59], Charmm [60], AmoebaBio [61], OPLS [62], Merck Molecular Force Field [63].

All three previous approaches described in the paragraphs above share the feature that the trajectory obtained by the user is in Cartesian coordinates. The web‐platform can handle this, but it can also handle an MD run in the normal‐mode reference. As anticipated, a normal‐mode approach may be useful to come up with an easier assignment of the vibrational features, and it is always possible to move from the Cartesian space to the normal‐mode one (and vice versa). The way to move from the Cartesian to the normal‐mode reference begins by calculating the Hessian of the potential energy, that is, the matrix of second derivatives of the potential energy with respect to the Cartesian coordinates, for the system in its equilibrium configuration (corresponding to the geometry of the potential energy minimum) and possibly oriented according to the Eckart frame. After that, the Hessian matrix must be mass‐weighted by the inverse of the product of the square roots of the atoms associated to the derivative coordinates. Eventually, one has to diagonalize the Hessian matrix, find its eigenvectors associated with non‐zero eigenvalues, and build a new matrix which has such eigenvectors as columns. We call C this matrix. It is an orthogonal matrix ($C^{-1}=C^{T}$) and it is possible to write

(15) $$\begin{array}{ll} \text{Q} & =C^{T}\text{x}\; \end{array}$$

(16) $$\begin{array}{ll} \text{P} & =C^{T}\text{v}\; \end{array}$$

where Q and x indicate the normal‐mode and Cartesian positions, respectively, and P and v represent normal‐mode momenta and Cartesian velocities, respectively. Once the input has been loaded, the user can get the spectrum in just a few seconds.

The web platform provides users with an accessible and installation‐free computational tool. The platform comprises two primary components: Python software for computing the power spectrum and a web interface facilitating user interaction and software execution. The web interface follows a client‐server architecture, where the client side, developed using HTML, CSS, and JavaScript, enables the users to input experiment parameters and visualize results. At the same time, the server side, implemented with Node.js and the Express framework, handles requests and executes the Python‐based computational engine. A key feature of SEMISOFT is its ability to start an independent instance of the software for each request, ensuring efficient multi‐user management. The user‐friendly interface simplifies the parameter entry process through drop‐down menus and tooltips, making it accessible even to non‐expert users. By leveraging a scalable server‐side infrastructure, SEMISOFT ensures efficient processing, making advanced vibrational spectroscopy techniques widely available without requiring local computational resources. The input dynamics files uploaded by the user are neither stored nor re‐used for any purpose.

**FIGURE 1:**
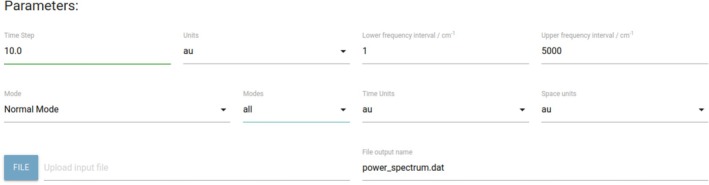
A screenshot of the web‐platform interface for inserting MD and power spectrum parameters.

Figure 1 reports the graphical user interface that allows one to input the parameters for calculating the power spectrum, as described in the following:

- “Time Step”: The user should introduce the time step employed in the molecular dynamics simulation;
- “Units”: These are the units of the Time Step adopted in the MD run. The user can choose between atomic units (au) and femtoseconds (fs);
- “Lower frequency interval/cm
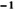
”: This is the initial frequency in cm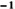 at which the power spectrum is calculated;
- “Upper frequency interval/cm
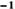
”: This is the final frequency in cm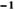 at which the power spectrum is calculated;
- “Mode”: This input is related to the space in which the molecular dynamics has been performed. It is possibile to choose between “Normal Modes” and “Cartesian”;
- “Modes”: If “Normal Modes” is selected in the previous entry, then it is possible to choose “sum”, which plots the sum of all $I_{j}(\omega )$ power spectra, or “all”, which displays all $I_{j}(\omega )$ at once without summing them, or one can explicitly pick one or more specific mode(s) to get the plot of the corresponding power spectra. If “Cartesian” is chosen in the previous entry, then it is possible to select the “Tinker” option for the molecular dynamics run with the Tinker package;
- “Time units”: These are the time units employed for velocities (or momenta) in the molecular dynamics file. The user can choose between atomic units (au), femtoseconds (fs), or picoseconds (ps);
- “Space units”: These are the space units employed for velocities (or momenta) in the molecular dynamics file. The user can choose between atomic units (au), nanometer (nm), and Angström (Å);
- “FILE” button: When pressed, the user is given the possibility to upload the molecular dynamics file from which to get the power spectrum;
- “File output name”: This is the name of the file where the power spectrum will be stored.

The necessity to specify the units of velocities and time evolution comes from the fact that different software may employ different units or the users themselves may have chosen to employ different units in their MD simulation. The standard MD file to be uploaded to the web‐platform is made of blocks of data, one for each step of the dynamics. Each block has a first line in which the number of atoms making up the system is reported followed by one line per atom: In the case of a Cartesian dynamics, the line starts with the atom label followed by the Cartesian coordinates and velocities; in the case of a normal mode dynamics: All normal mode momenta are listed. A particular case is related to the Tinker package, which is a very popular suite of codes to run MD using the most popular force fields. It has a specific output and by checking the “Tinker” box, the web‐platform is instructed to read from an MD input file previously generated with Tinker. Velocity units (Å/ps) used in Tinker must be anyway specified in the graphical interface by the user for the correct functioning of the web‐platform. Finally, once the spectrum has been generated, from the plot screen, it is possible to zoom in and out, download the raw data of the spectrum, and download the image.

The web‐platform is equipped with an internal error check that prints on screen an error message to inform the user if the input dynamics file is not compatible with the selected “Mode” and “Modes” options. The user is still required to insert the correct units in agreement with the source dynamics file.

## 3 Results and Discussion

The web‐platform can be found at http://semisoft.unimi.it/, and it comes with three examples to help the user practice it. Here, to demonstrate the capabilities of the web‐platform, we investigate two systems: Aniline microsolvated by a single water molecule, and the isolated deoxyguanosine molecule. We present a discussion of some of the vibrational features of these two systems based on the use of the web‐platform. Furthermore, we compare different options available in the platform, which is a good way to illustrate its main features. The trajectory files employed as input for the studies presented here are readily downloadable through the “Download examples” button on the platform website.

The aniline‐water system is studied by means of an ab initio “on‐the‐fly” Cartesian dynamics generated with the NWChem (v. 7.2.1) software. The trajectory was previously run for a different kind of study before the release of the web‐platform (see Reference [20].). The level of electronic theory is DFT with B3LYP functional and aug‐cc‐pVDZ basis set. Initial conditions are chosen to be the equilibrium geometry at the global minimum and atomic velocities contributing a total energy equal to the harmonic zero‐point energy. The dynamics is evolved for a total of 25,000 a.u. (about 0.6 ps) and each step is of 10 a.u. Velocity units are in a.u.

The interest in a system such as microhydrated aniline is related to the study of the mechanisms of solvation of organic matter and reactivity in hydrogen‐bonding solvents. In the hydrogen bond of the equilibrium configuration, the oxygen acts as a hydrogen donor, and the nitrogen of aniline serves as a hydrogen acceptor. The web‐platform is capable of producing the entire vibrational power spectrum from the autocorrelation of atomic velocities along the dynamics. We start by showing a comparison between the spectrum based on the original ab initio trajectory as described above, and the one obtained upon transformation of the trajectory to the 45‐dimensional normal mode space (see Equations (15) and (16)). This demonstrates that the platform can work both in the Cartesian and normal mode spaces.

**FIGURE 2:**
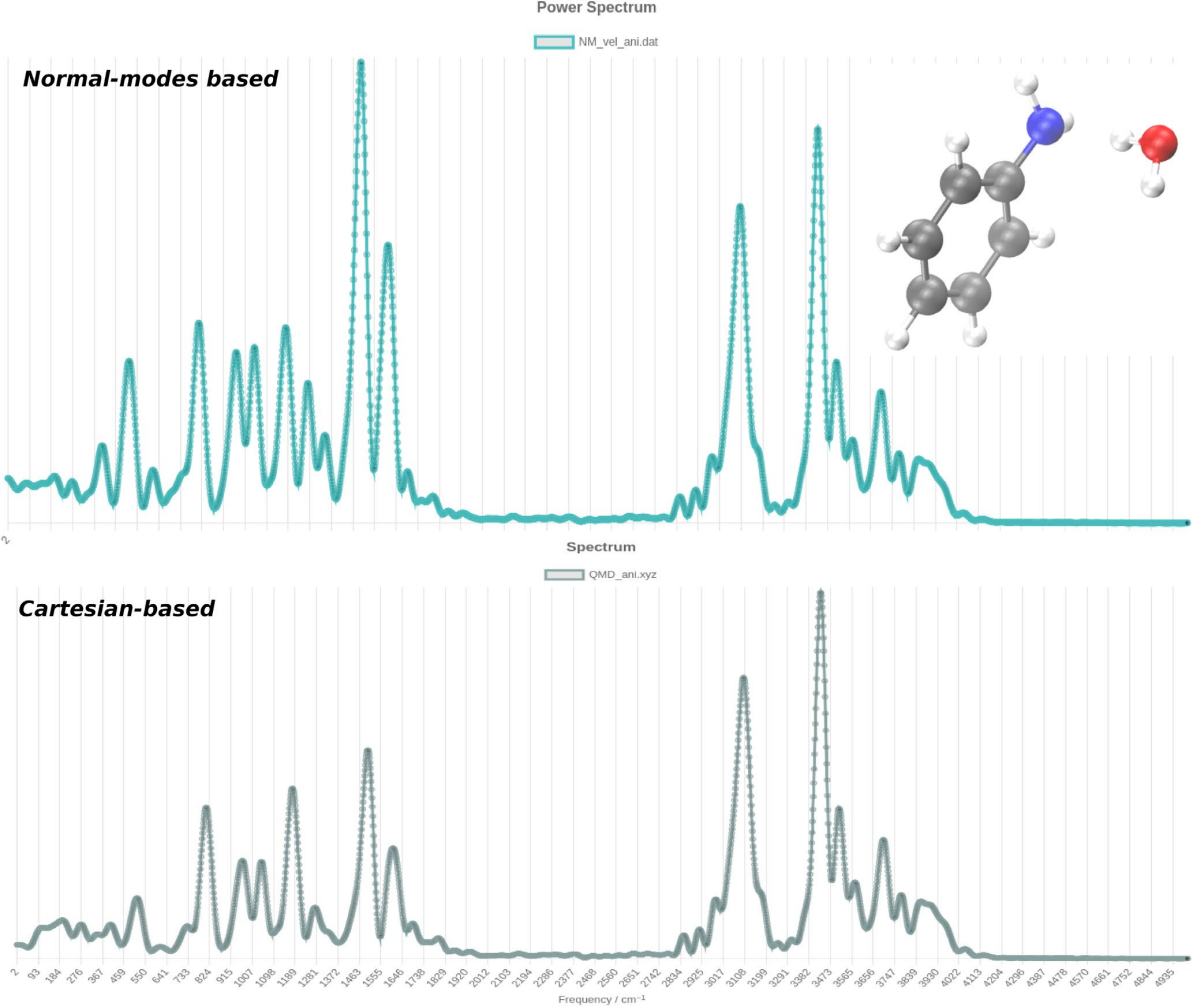
Power spectrum for monohydrated aniline: Spectrum based on a Cartesian trajectory (bottom panel), and obtained from the same trajectory in normal mode space and with “Modes” option “sum” in the web platform (upper panel).

The spectrum in Figure 2 is clearly divided into a low‐frequency part and a high‐frequency range. The high‐frequency spectrum is exactly superimposable in the two cases, while in the low‐frequency range, some minor differences are present. The latter are due to the fact that the C matrix employed to move from the Cartesian space to the normal mode one is rigorously defined only at the equilibrium geometry, but employed as is for geometries along the trajectory. The result is that a small amount of rotation is introduced with perturbation of the lower frequency vibrations. In spite of this, Figure 2 demonstrates that a normal mode‐based analysis of the spectroscopic signals is not only doable with the web‐platform but also reliable.

As anticipated in the Introduction, it is important to be able to assign the several spectral signals with good confidence. Of particular interest for the aniline‐water system is the high‐frequency region. From Figure 2, one can notice that the CH‐stretch band extends from about 3,000 cm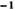 to about 3,200 cm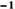. Above 3,400 cm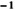 several signals are present. They can be due to the two (symmetric and asymmetric) fundamental NH stretches of aniline, or the two OH stretches (symmetric and asymmetric) of the water molecule, or even some sum‐of‐lower‐frequency signals which are expected to appear in a classical power spectrum based on a single trajectory. In this paper, we focus on the CH‐stretch band, where five fundamental frequencies are present. The CH‐band for aromatic compounds is usually found at 3,000–3,200 cm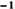. We notice this is at a higher frequency than for other compounds, due to the presence of the aromatic ring or electronegative substituents. In Figure 2 the band is characterized by a maximum at about 3,100 cm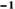 with three side peaks at a lower frequency. The side peaks can be due to sum‐of‐frequencies of modes in the 1,400–1,600 cm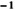 range. Furthermore, it is not possible to assign the five CH fundamentals within the band.

To assign more precisely the frequencies to the five fundamentals, we exploit two features of the web‐platform for spectra based on a dynamics in the normal‐mode space. The first one is that one can plot the spectrum relative to more than one mode at the same time. The other feature is that, starting from such a multi‐mode spectrum, it is possible to hide some of the contributions and further decompose the spectral bands. The latter characteristic is particularly important in systems such as aniline‐water, which are characterized by strongly coupled modes. As anticipated, when this happens, the single‐mode spectra present several peaks due to the coupling to the other modes.

**FIGURE 3:**
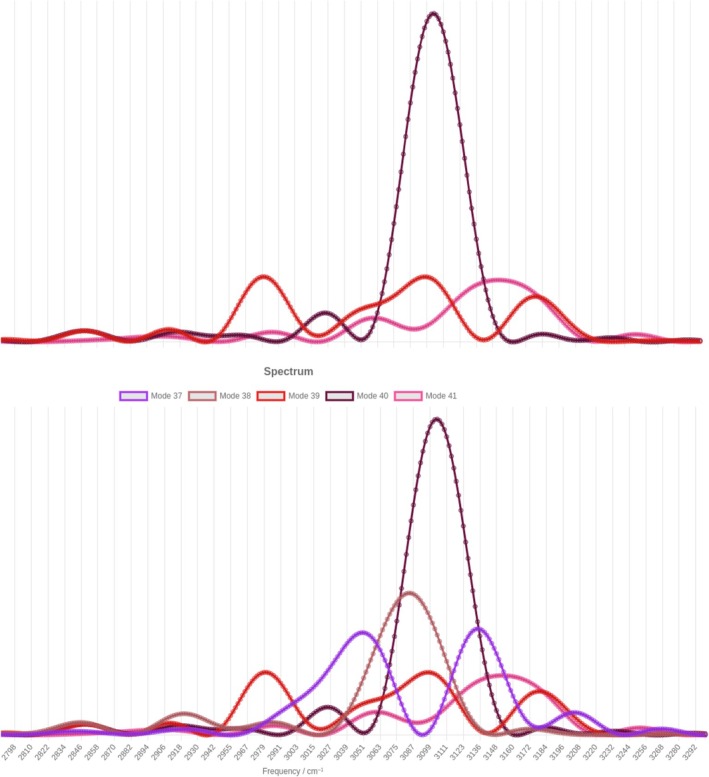
CH‐stretch band for the aniline‐water system. Complete CH‐stretch modes (bottom panel). Same spectrum but with the two lower frequencies CH stretches, which have been hidden (upper panel).

Figure 3 reports the five calculations related to the CH stretches (i.e., $I_{n}(\omega )$ for mode number n between 37 and 41) in the bottom panel. It is evident that the five modes are all coupled together. Therefore, to assign the peaks, it is necessary to hide spectra one‐by‐one and, for each calculation, determine the main peaks and those due to coupling. As an example, in the upper panel of Figure 3 we hide $I_{37}(\omega )$ and $I_{38}(\omega )$. This allows us to determine the three main peaks for modes 39–41, which are located in ascending order of frequency. Furthermore, it is easy to identify the peaks due to coupling: Each spectrum in the upper panel presents several peaks, mode 39 being the one with peak intensities more similar to each other. By means of this peak‐by‐peak analysis permitted by the platform, we are able to assign the frequencies of the CH‐stretch band with more confidence. Numerical results are reported in Table 1. The web‐platform provides an excellent tool to help assign accurately the signals, and the same procedure previously illustrated can be successfully employed for other regions of the spectrum.

**TABLE 1:** Assignment of vibrational frequencies for the CH‐stretch band of monohydrated aniline. Values are in cm.

Mode	37	38	39	40	41
$\nu_{NM}$	3,049	3,084	3,098	3,104	3,153

Then, we move to the second system under consideration, that is, the deoxyguanosine molecule. This molecule is made of 32 atoms and 90 vibrational degrees of freedom. It is unnecessary to stress its importance in biochemistry, being one of the constituents of DNA. It is made of a purine nucleobase and a ribose sugar, so the power spectrum is expected to show the fingerprints of CH‐stretches, NH‐stretches, and OH‐stretches in the high‐frequency region. Given the large dimensionality of deoxyguanosine, the electronic structure problem is tackled by means of the polarizable force field AMOEBABIO18 [61], and the dynamics is performed with the Tinker molecular modeling package [64]. The dynamics is made of steps of 0.2 fs for a total of 3,000 steps (i.e., a total evolution time of 600 fs). The web‐platform is able to read the Tinker output (selection “Modes”‐> “Cartesian” and then check the “Tinker” box) and velocities are printed in units of Å/ps. The initial conditions are set at the equilibrium geometry of deoxyguanosine, while velocities are determined in a way that each j‐th mode contributes a kinetic energy of $\omega_{j}⁄2$, so that the total energy equals the harmonic zero‐point one.

**FIGURE 4:**
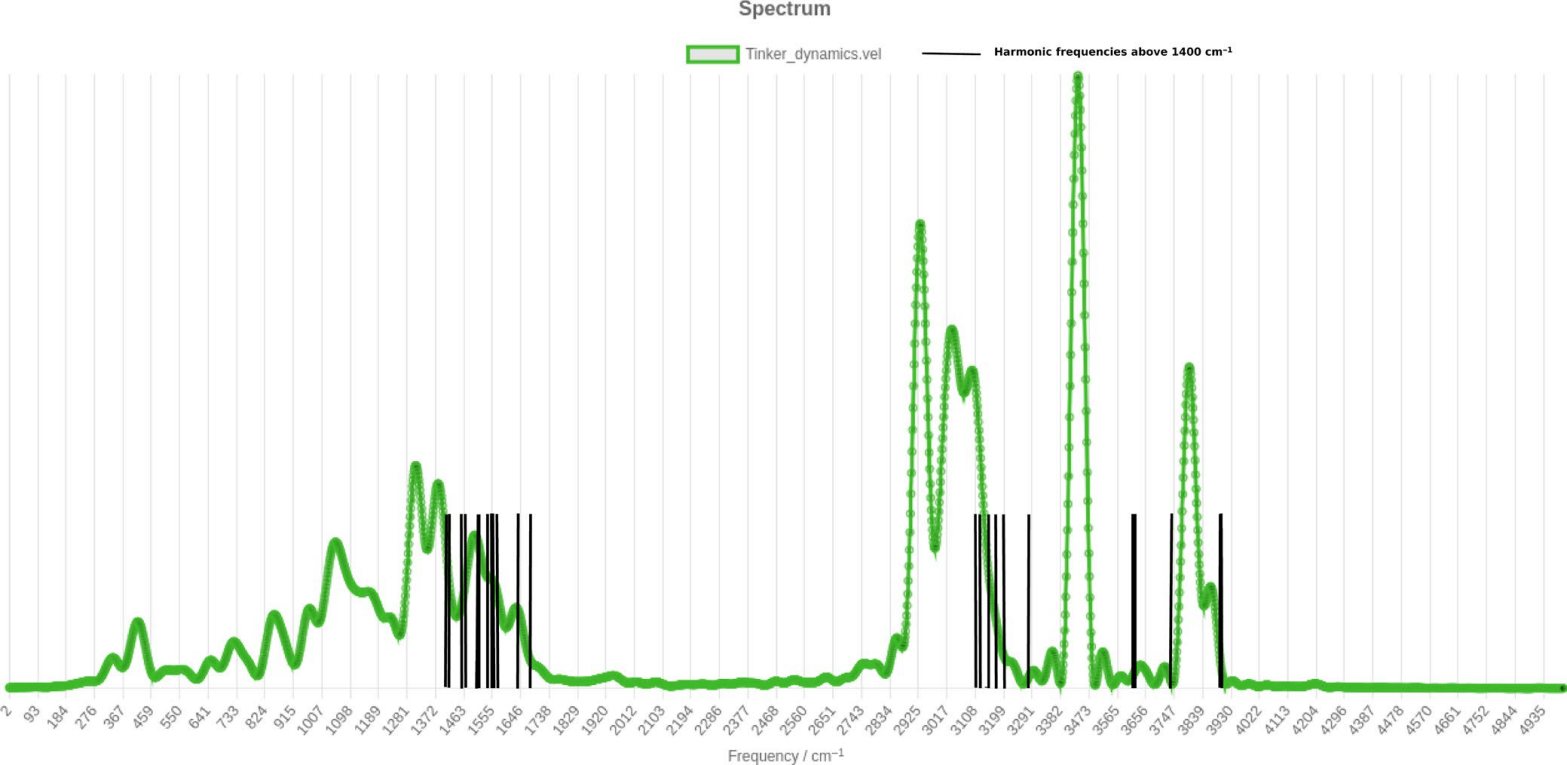
Power spectrum of deoxyguanosine from a molecular dynamics trajectory generated using the AMOEBABIO18 polarizable force field (green). Harmonic frequencies above 1,400 cm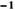 are reported with black sticks.

Figure 4 shows the power spectrum obtained from the web‐platform using the Tinker dynamics output file with no post processing. We notice that in the high‐frequency region, three bands are clearly visible: They are the CH‐stretch band, the NH‐stretch band, and the OH‐stretch band in ascending order of frequency. Specifically, in the case of deoxyguanosine there are 8 CH‐stretch modes, 3 NH‐stretch modes, and 2 OH‐stretch modes. As in the case of monohydrated aniline, one should try to decompose the spectrum using the normal‐mode space to come up with a more detailed assignment. Here we limit ourselves to the demonstration that the web‐platform is suitable for working with common biochemical software, providing a new tool to the biochemical community. Table 2 reports a comparison between harmonic and anharmonic frequencies obtained from the classical power spectrum for the high frequencies of deoxyguanosine. Without a mode‐by‐mode spectral analysis, we choose to indicate just the range of the three stretch bands. However, this is sufficient to demonstrate that a scaled harmonic approach is inappropriate for this system, since the different bands are characterized by a different amount of anharmonicity. This effect is visible by comparing the SEMISOFT spectrum in Figure 4 with the stick spectrum of harmonic frequencies reported therein.

**TABLE 2:** Harmonic and anharmonic estimates of frequency ranges for the three stretch bands of deoxyguanosine. Values are in cm.

	CH‐stretch band	NH‐stretch band	OH‐stretch band
Harmonic	3,100–3,275	3,610–3,730	3,890–3,892
Anharmonic	2,900–3,140	3,400–3,470	3,770–3,880

## 4 Summary and Conclusions

In this paper, we have introduced a web‐platform to calculate the Fourier transform of autocorrelated data. The web‐platform is free to use and can be found at the following web address: http://semisoft.unimi.it/. Our simulations have focused on two systems: Monohydrate aniline and deoxyguanosine. We have shown the potential of the web‐platform in elaborating spectra starting from both a Cartesian dynamics and the dynamics obtained by transforming the Cartesian one to the normal mode coordinates. Exploiting the platform's capability to compute single‐mode spectra with the time averaging technique, the calculation based on normal modes provides spectra that are easier to assign. The main vibrational characteristics of the two systems have been correctly identified, and the focus has been on the high‐frequency range of fundamentals. We remind the reader that we calculated power spectra originating from velocity autocorrelation functions, so selection rules do not apply in these cases. To include selection rules, one should work with the dipole autocorrelation function. The web‐platform can handle both types of calculations.

We think the web‐platform could be a valuable tool for physics, chemistry, and biology communities. For instance, in biochemistry vibrational spectra (IR or Raman) are hardly ever accompanied by theoretical calculations and, when they are, the adopted approach is very often an inappropriate scaled harmonic one. In the past, three of us have studied the anharmonic spectrum of a G‐quadruplex [65], providing a new level of theoretical calculations to assist experimental assignments. Other examples of investigations that could be tackled with the help of the web‐platform, include studies to pinpoint atomic coupling in molecules by calculating the power spectra of a parametrically tuned Hamiltonian MD dynamics [66], and the possibility to distinguish the type of molecular adsorption of NO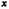 compounds or water molecules on titania surfaces [67, 68]. Overall, the web‐platform introduced here aims at making higher‐level calculations and more accurate assignments routinely doable.

To conclude we would like to remark that the web‐platform has a general purpose and it is not limited to the calculation of vibrational spectra. Autocorrelated data play an important role in many other fields, even not related to chemistry, and the Fourier transform of such autocorrelation functions can provide valuable information on recurrent patterns of historical data. For instance, in finance, autocorrelation functions are employed to determine how much of an impact historical prices of a security or a stock have on its future price. In medicine, they may be employed to visualize blood flow. In music, autocorrelation functions are used in relation to instrument tuners. In astronomy, they allow to determine of the frequencies of pulsars (rapidly rotating neutron stars). These are just a few examples of the wide range of applications of the web‐platform presented here.

## Conflicts of Interest

The authors declare no conflicts of interest.

## Supporting information


**Data S1.** Supporting Information.

## Data Availability

The data that support the findings of this study are available from the corresponding author upon reasonable request.
