# Supplementary material for: Vibrational Spectroscopy Through Time Averaged Fourier Transform of Autocorrelated Molecular Dynamics Data: Introducing the Free SEMISOFT Web‐Platform
Source: J Comput Chem. 2025 May 3;46(12):e70118. doi: 10.1002/jcc.70118 (PMC12049240; doi:10.1002/jcc.70118)
Supplement: Supplementary file 1 — Data S1. Supporting Information. [file JCC-46-0-s001.zip › video caption.docx]

The legend is:

 "Demo video for use of the SEMISOFT platform"
